# Drawing the line

**DOI:** 10.1186/s12915-015-0190-9

**Published:** 2015-10-02

**Authors:** Emma Saxon

**Affiliations:** BMC Biology, BioMed Central, 236 Gray’s Inn Road, London, WC1X 8HB UK

## Abstract

The pea aphid *Acyrthosiphon pisum* is an agriculturally important pest of leguminous plants including peas and broad beans. The widespread use of chemical pesticides impacts heavily on the environment, and increases pesticide-resistant pea aphid populations, so alternative strategies are being actively sought. *Pseudomonas syringae* bacteria are known to infect and kill the pea aphid, and offer a possible control strategy. In this study, the authors measured the effects of injecting *P. syringae* on the survival of pea aphid populations at 24 and 48 hours. The pea aphid population was killed more rapidly (98 % after 24 hours) with a higher concentration of injected bacteria than in the control or with lower concentrations, indicating that a *P. syringae*-based control strategy may be a useful alternative to conventional pesticides.

## Comment

**Fig. 1:**
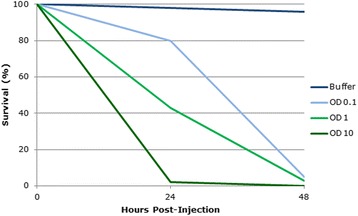
Percentage survival of *Acyrthosiphon pisum* (pea aphid) populations injected with different concentrations of *Pseudomonas syringae* bacteria over 48 hours

Line graphs can hide complexity in the relationship between two variables if the data are sparse. The authors of this graph (Fig. 1) injected populations of pea aphids (*Acyrthosiphon pisum*) with a laboratory-grown strain of *Pseudomonas syringae* bacteria known to have potential as an insecticidal agent. Insect survival was measured at 0, 24, and 48 hours after injection with the bacterial cultures grown to different optical densities (ODs), as compared with a buffer control, and plotted on the graph, with lines drawn between the points to indicate the rate of change in insect survival. While the points on the graph indicate that most of the insects infected with the bacterial culture at an OD of 10 died within the first 24 hours, and most of those infected at an OD of 0.1 died between 24 and 48 hours post-injection, the lines joining these points suggest that the rate of death was constant over these 24-hour periods.

However, drawing a line through just three points, as the authors have done, is unlikely to be an accurate representation of the relationship between percent survival and hours post-injection. It may be that the survival of insects injected with the bacteria at an OD of 10 decreased more rapidly; for example, the graph indicates that survival was 45 % after 12 hours, but it could equally have been 20 %, which is a considerably shorter time frame than this graph suggests. This could affect the authors’ conclusions: in this case, the critical difference may not be the total number of aphids killed by 24 hours post-infection, but how many were killed within 6 or 12 hours, as pea aphids can very rapidly cause damage on crop plants. More data from intermediate time points are clearly required to describe the relationship.

